# Retinol binding protein 4 abundance in plasma and tissues is related to body fat deposition in cattle

**DOI:** 10.1038/s41598-019-44509-4

**Published:** 2019-05-30

**Authors:** Yinuo Liu, Elke Albrecht, Dirk Dannenberger, Harald M. Hammon, Christa Kuehn, Helga Sauerwein, Runjun Yang, Zhihui Zhao, Steffen Maak

**Affiliations:** 1 College of Animal Science, Jilin University, Changchun, Jilin, 130062 P.R. China; 2 Institute of Muscle Biology and Growth, Leibniz Institute for Farm Animal Biology (FBN), 18196 Dummerstorf, Germany; 3 Institute of Nutritional Physiology “Oskar Kellner”, Leibniz Institute for Farm Animal Biology (FBN), 18196 Dummerstorf, Germany; 4 Institute of Genome Biology, Leibniz Institute for Farm Animal Biology (FBN), 18196 Dummerstorf, Germany; 5 Institute for Animal Science, Physiology and Hygiene Unit, University of Bonn, 53115 Bonn, Germany; 6 College of Agriculture, Guangdong Ocean University, Zhanjiang, 523088 P.R. China

**Keywords:** Cellular imaging, Fat metabolism

## Abstract

Retinol binding protein 4 (RBP4) facilitates the transport of retinol in the body but is also an adipokine and fatty acid transporter. Our study was aimed at investigating the associations between RBP4 abundance and fat deposition in cattle. Blood samples of 246 crossbred bulls were taken at 8 months of age and at slaughter at 18 months of age for the determination of RBP4, hormone levels, and fatty acid composition. Significant correlations between plasma RBP4 abundance at 8 months of age and carcass traits at 18 months of age were detected (e.g., r = 0.3; P < 0.001 to carcass fat). Furthermore, RBP4 abundances in the plasma and subcutaneous fat were higher (P < 0.05) in bulls with increased fat deposition, whereas the liver RBP4 expression was not (P > 0.05). Retinol binding protein 4 was immunohistochemically localized in or close to adipocytes within muscle and adipose tissue and in liver stellate cells but not in hepatocytes. Overall, our results indicate that increased RBP4 levels were associated with increased fat deposition and altered fatty acid composition, but not with altered glucose tolerance, in crossbred bulls. Moreover, our results suggest that adipose-tissue-derived RBP4 may contribute to the circulating RBP4 level.

## Introduction

Retinol binding protein 4 (RBP4) is the only circulating member among four proteins of this family and facilitates the transport of retinol (vitamin A) from the liver to the peripheral organs^1,2^. Many cellular processes depend on the availability of retinol, in particular the regulation of gene expression, through binding to nuclear transcription factors involved in the maintenance of epithelial surfaces, immune competence, and reproduction^3^, thus making RBP4 an essential factor for the organism. Retinol transport was long assumed as the only function of RBP4^4^. More recently, RBP4 was shown to also act as an adipokine that is involved in the modulation of insulin sensitivity in mice and humans^5^. However, data in humans revealed conflicting results regarding relationships between circulating RBP4, its gene expression and obesity or insulin resistance (reviewed by Kotnik *et al*.^6^). This may indicate differences between rodent models and humans in this respect^6^. Moraes-Vieira *et al*.^7^ demonstrated that increased RBP4 activates innate immunity, with a subsequent adaptive immune response leading to the inflammation of adipose tissue. This finally results in systemic insulin resistance. There is still controversy, however, about the origin of the increased circulating RBP4 levels during insulin resistance^8^.

The function of RBP4 may be even broader than currently known. As recently demonstrated by Perduca *et al*.^9^, RBP4 can bind and transport fatty acids. Furthermore, RBP4 functions as a hepatokine and mediates the effects of the molecular clock on glucose metabolism in mice^10^.

Beside hepatocytes as the main source of RBP4, it is expressed in adipocytes, macrophages, myocytes and further tissues and cells^5,11,12^. Only recently, hepatocytes were identified as the principal source of circulating RBP4 in mice^8^.

Although bovine RBP4 was characterized at the biochemical level decades ago^2,13^ its physiological role is still not well understood. Abd Eldaim *et al*.^14^ determined circulating RBP4 concentrations of approximately 45 µg/mL in the plasma of non-pregnant, non-lactating cows with quantitative western blots. The plasma RBP4 concentrations were similar in pregnant cows at 3 to 6 d before parturition but decreased immediately after parturition to ~25 µg/mL^14^. RBP4 was obviously released into colostrum but was almost undetectable in milk, whereas the plasma levels then returned to values observed in control cows. RBP4 protein was also shown to increase in subcutaneous fat (SCF) with age when comparing the SCF proteome in 12- and 15-month-old steers^15^.

The mRNA abundance of *RBP4* was related to the intramuscular fat (IMF) content in M. longissimus of cattle^16^. De Jager *et al*.^16^ identified *RBP4* as a member of a gene set involved in intramuscular adipocyte lipid metabolism in Piedmontese × Hereford and Wagyu × Hereford crosses. Furthermore, the analysis of Brahman cattle also revealed a positive correlation between *RBP4* expression and intramuscular fat deposition^16^. Additionally, several studies found an inverse relationship between vitamin A supply and IMF content in different cattle populations^17–20^. However, the magnitude of this relationship is unclear, and the physiological mechanism behind this phenomenon remains unaddressed. Due to the recently proposed functions of RBP4 beyond retinol transport, such as fatty acid transport^9^, its potential involvement in fat deposition and composition in cattle is of interest. Consequently, RBP4 may be a candidate factor for the variation in carcass composition in cattle and the fatty acid composition of beef. Thus, our study was aimed at the elucidation of *RBP4* expression at mRNA and the protein level in bovine tissues and relationships between circulating RBP4, carcass parameters, fatty acid composition and hormone concentrations in the plasma of phenotypically diverse cattle. An F_2_-cross, generated from Charolais bulls and German Holstein cows in the founder generation^21^, was the experimental basis for this investigation. The founder breeds represent cattle of comparable stature but different growth rates and accretions of protein and fat. Charolais bulls grow faster and have higher protein accretion than German Holstein bulls, which accrete more fat in the carcass^22^. Bulls of the F_2_ generation varied widely in carcass fat deposition, providing an excellent model to study the mechanisms involved in carcass composition and, in particular, the role of RBP4 as one potential factor. To this end, we used reverse transcription – quantitative PCR (RT-qPCR) for the measurement of tissue-specific mRNA abundance. The RBP4 protein amount was determined semi-quantitatively in the serum and tissues by western blot analyses of protein extracts with antibodies for RBP4. Antibodies were tested for specificity, and one of the antibodies was also used to visualize the tissue localization of RBP4.

## Results

### RBP4 expression is different in liver and adipose tissue of bulls with high or low carcass fat deposition

The abundance of *RBP4* mRNA was compared between two groups of bulls differing in their total amount of carcass fat selected from 246 F_2_ generation bulls of a Charolais × Holstein cross, as described in the Materials and Methods section. The high carcass fat group (HCF) accreted 94.2 ± 6.4 kg fat in the carcass, and the low carcass fat group (LCF) accreted 36.0 ± 6.4 kg. Higher (P < 0.01) concentrate consumption (HCF: 7.5 ± 0.6 kg/d vs. LCF: 5.9 ± 0.7 kg/d), residual feed intake (HCF: 7.0 ± 4.8 vs. LCF: −3.5 ± 6.1), and feed conversion ratio (HCF: 70.2 ± 3.6 MJ ME/kg vs. LCF: 65.4 ± 6.2 MJ ME/kg) were recorded for bulls with higher carcass fat deposition. Further phenotypic traits of both groups are presented in Supplemental Table 1 and were originally described by Liu *et al*.^23^.

**Figure 1:**
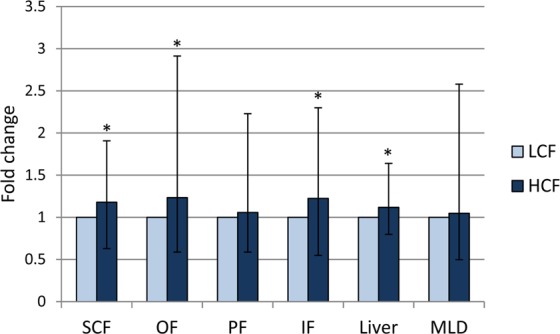
Fold changes of *RBP4* mRNA abundance in different adipose tissues, liver and muscle of high carcass fat (HCF, n = 20) compared to low carcass fat (LCF, n = 18) bulls. Data were normalized to beta-2-microglobulin (*B2M*) and ubiquitously expressed transcript (*UXT*) for adipose tissues, *UXT* and ribosomal protein S9 (*RPS9*) for liver tissue, and topoisomerase II beta (*TOP2B*) and *B2M* for muscle tissue and calculated using the REST software (Version 2.0.13, QIAGEN, Hilden, Germany). Graphs show fold changes of expression of HCF vs. LCF bulls with standard errors calculated by REST software^45^. * indicate significant differences between groups with P < 0.05. SCF – subcutaneous fat, PF – perirenal fat, OF – omental fat, IF – intestinal fat, MLD – Musculus longissimus dorsi.

The mRNA abundance of *RBP4* was slightly upregulated in subcutaneous (SCF), omental (OF), and intestinal fat (IF) and in the liver of bulls with high carcass fat deposition (HCF; 1.2-, 1.2-, 1.2-, and 1.1-fold, respectively, P < 0.05, Fig. 1) compared to LCF bulls. In perirenal fat (PF) and muscle tissue, the *RBP4* mRNA levels were similar in both groups (P > 0.05). Normalized mRNA values of different types of tissues were obtained using different reference genes (see Materials and Methods). Therefore, those values are not directly comparable between liver, muscle and adipose tissues. However, considering the raw C_p_-values and amplification efficiency, the data indicate the highest *RBP4* expression in the liver, slightly lower but similar expression in SCF and PF, and a clearly lower expression in OF, IF, and muscle tissue (Supplemental Table 2).

The RBP4 protein was detected by western blot in all adipose tissues and the liver, but not in muscle tissue, although *RBP4* mRNA was measured in each muscle sample from both groups of bulls. The specificity of both antibodies was tested in advance and concordantly revealed a protein band for recombinant bovine RBP4 at ~20 kDa (Supplemental Fig. 1, lanes 5–7). The detected protein band in bovine plasma, SCF, and the liver was slightly smaller than that of recombinant protein and appeared at ~18 kDa (Supplemental Fig. 1, lanes 1–3, respectively). Both antibodies generated similar results in the bovine plasma, adipose tissue, and liver. No specific band could be detected in the muscle tissue (lane 4). The specificity of the observed bands could be verified by blocking specific bindings by preincubation of the antibodies with recombinant protein. Only a few unspecific bindings were found. The identity of the band of interest was verified by mass spectrometry (data not shown). The band of interest at ~18 kDa actually contained signatures specific for RBP4; thus, both antibodies were used for the quantification of RBP4 protein abundance.

**Figure 2:**
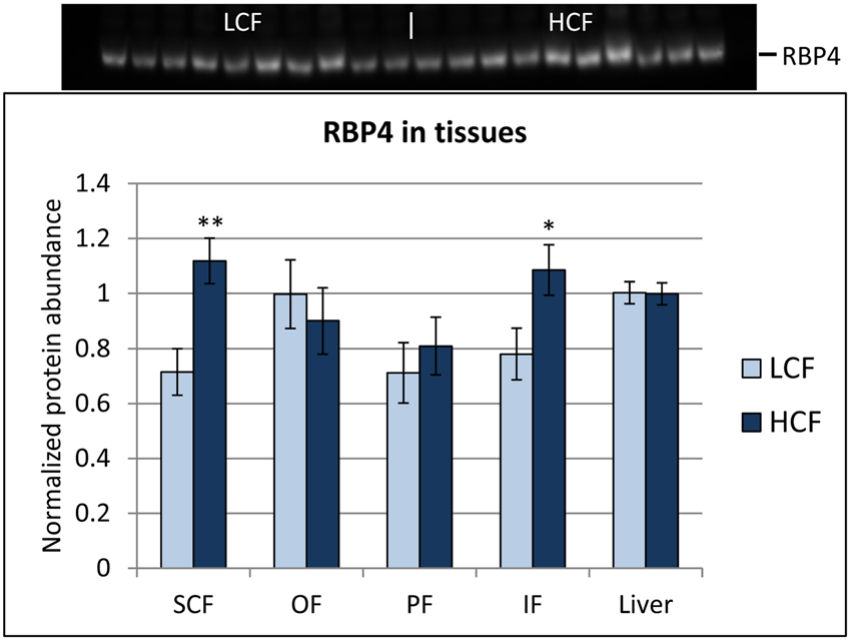
Relative protein abundance of RBP4 in different adipose tissues and the liver of high carcass fat (HCF, n = 20) and low carcass fat (LCF, n = 18) bulls at an age of 18 months. Data were normalized to total protein in each lane and are expressed as LSmean ± SE. * and ** indicate significant differences between groups with P < 0.05 and P < 0.01, respectively. A representative blot image of the liver, cropped to the respective band, is shown above the graph. The complete blot and a blot of SCF are shown in Supplemental Fig. 2. SCF – subcutaneous fat, OF – omental fat, PF – perirenal fat, IF – intestinal fat.

The protein abundance was determined as band volume normalized with total protein in the respective lane as a loading control and divided by the mean intensity of all signals in the blot to enable a blot-to-blot comparison. Values are referred to as normalized protein abundance (Fig. 2, Supplemental Fig. 2). The normalized RBP4 protein abundances were higher in SCF and IF of HCF compared to LCF bulls, in concordance with the mRNA data. There was no difference in RBP4 protein abundance between the groups in PF, OF, and the liver. A comparison of protein abundances among different tissues was not possible with this method because the tissues were treated differently and have different protein composition. This leads to altered staining efficiency and the appearance of total protein (see Supplemental Fig. 2a,b). However, a test with equally treated protein extracts indicated similar RBP4 concentrations in all adipose tissues and in the liver (Supplemental Fig. 3). Thus, it cannot be claimed that the higher *RBP4* mRNA abundance in the liver and SCF is reflected by higher protein concentrations in the respective tissues. A significant correlation between mRNA and protein abundance was found in OF (r = 0.318; P < 0.05; n = 38) but not in other tissues.

### RBP4 protein is localized in adipocytes, liver stellate cells and macrophages

**Figure 3:**
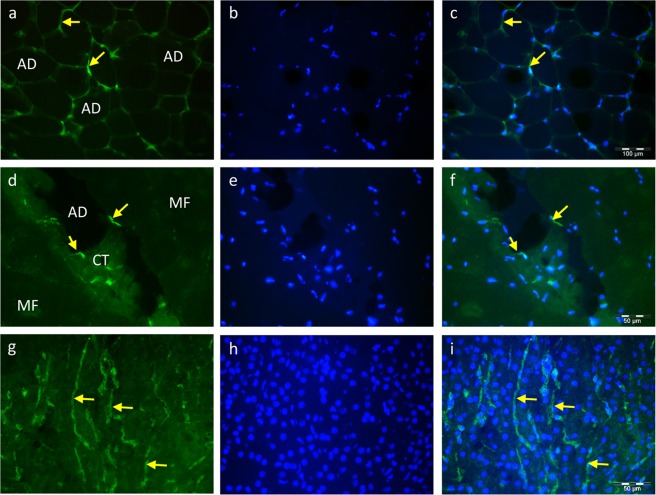
Cellular localization of RBP4 in subcutaneous fat (**a**–**c**), muscle tissue (**d**–**f**), and bovine liver (**g**–**i**) by immunohistochemistry. (**a**,**d**,**g**) RBP4 detection (secondary antibody: Alexa Fluor 488 conjugated goat anti rabbit IgG, green), (**b**,**e**,**h**) nuclear staining with Hoechst 33258 (blue), (**c**,**f**,**i)** merged images. Arrows indicate stained cells with cytoplasmic RBP4 localization. Images were equally enhanced in contrast. AD – adipocyte, CT – connective tissue, MF – muscle fibre.

**Figure 4:**
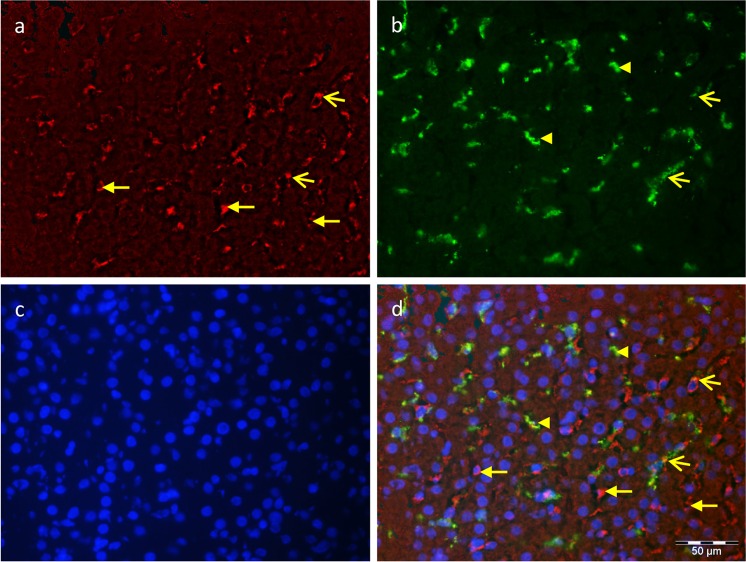
Cellular localization of RBP4 (**a**, MFP 590 conjugated goat anti rabbit IgG, red) and the macrophage marker CD163 (**b**, Alexa Fluor 488 conjugated goat anti mouse IgG, green) in bovine liver. (**c**) Nuclear staining with Hoechst 33258 (blue); (**d**) merged image. Open arrows indicate co-localization, closed arrows indicate cells exclusively immunoreactive for RBP4, and arrow heads indicate exclusively CD163 immunoreactive macrophages.

Immunohistochemistry was applied to localize RBP4 in different tissues. In subcutaneous adipose tissue (Fig. 3a–c), the fluorescence signal was associated with adipocytes, but further cell types situated between adipocytes are likely to contribute to RBP4 immunofluorescence. In muscle tissue, RBP4 signals could only be detected in the small cells within the connective tissue in close proximity to intramuscular adipocytes (Fig. 3d–f). There were no fluorescence signals for RBP4 within muscle fibres. A clear signal for RBP4 was observed in the cytoplasm of liver cells, which were obviously not hepatocytes. Co-localization with cells having a characteristic shape and auto-fluorescence led us to conclude that at least a portion of the RBP4-positive cells were stellate cells (Fig. 3g–i). To test whether macrophages also contributed to the observed RBP4 signals, double labelling with CD163, a macrophage marker, was performed. Both signals were co-localized in only a few cases (Fig. 4), indicating RBP4 containing macrophages. Most cells were either RBP4 or CD163 positive.

### Higher levels of circulating and adipose tissue RBP4 in bulls are associated with increased fat accumulation

**Figure 5:**
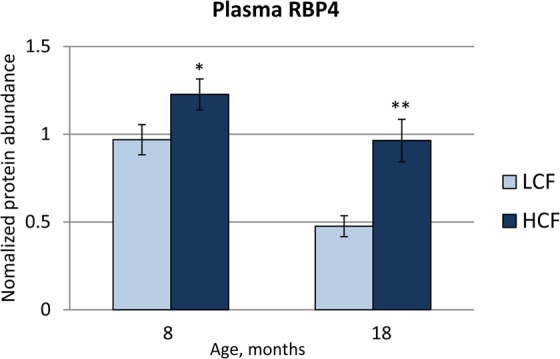
Abundance of RBP4 protein in plasma of HCF and LCF bulls at 8 months and 18 months of age. Data are expressed as LSmean ± SE. * and ** indicate significant differences between groups with P < 0.05 and P < 0.01, respectively. HCF – high carcass fat, n = 20; LCF – low carcass fat, n = 18. A representative blot image is shown in Supplemental Fig. 4.

RBP4 is a secreted protein that can be found in circulation^14^. To test whether the plasma RBP4 levels are related to body composition or to the amount of accumulated fat in the body, the RBP4 levels were determined in all available plasma samples of the F_2_-crossbred bulls (example in Supplemental Fig. 4). Due to the loss of samples and technical reasons, the plasma RBP4 levels were measured in 216 bulls at 8 months of age and in 220 bulls at 18 months of age. Additionally, RBP4 abundance in SCF was measured in all 246 bulls. Pearson correlation coefficients between body composition traits and RBP4 levels were analysed and are presented for the selected traits in Table 1. Plasma RBP4 levels at 8 months of age were significantly correlated with fat deposition traits at slaughter (Table 1; e.g., r = 0.3; P < 0.001 to cold carcass fat). However, those relationships were not observed at 18 month of age, except for a significant correlation between plasma RBP4 and liver weight (Table 1). Plasma RBP4 levels were significantly higher (P < 0.05) in HCF compared to LCF subsets of bulls at both ages (Fig. 5). An estimation of actually circulating RBP4 concentrations was done with western blot analysis of plasma samples without albumin depletion of both ages together with a dilution series of recombinant RBP4 protein on the same blots (Supplemental Fig. 5). The calculated values varied between 22 and 83 µg/mL and confirmed significantly higher concentrations at 8 months than at 18 months of age in both groups (HCF µg/mL: 56.4 ± 2.9 and 45.7 ± 2.9; LCF µg/mL: 49.8 ± 3.1 and 40.5 ± 3.1; at 8 and 18 months respectively).

**Table 1:** Pearson correlation coefficients between traits of F_2_-generation bulls at 18 months of age and RBP4 protein abundances in plasma at 8 and 18 months of age and in subcutaneous fat at 18 months of age.

Trait	RBP4 in plasma (8 months, n = 216)		RBP4 in plasma (18 months, n = 220)		RBP4 in SCF (18 months, n = 243)	
	r	P - value	r	P - value	r	P - value
Body weight, kg	0.097	0.157	0.062	0.360	**0**.**126**	**0**.**049**
Liver, kg	**0**.**168**	**0**.**014**	**0**.**254**	<**0**.**001**	**0**.**259**	<**0**.**001**
Cold carcass weight, kg	0.091	0.185	0.062	0.359	**0**.**177**	**0**.**006**
Cold carcass fat, kg	**0**.**303**	<**0**.**001**	0.104	0.123	**0**.**166**	**0**.**009**
Perirenal fat, kg	**0**.**151**	**0**.**027**	−0.080	0.236	0.085	0.185
Intestinal fat, kg	0.093	0.173	−0.016	0.813	−0.037	0.566
Omental fat, kg	0.111	0.103	0.002	0.978	0.055	0.396
Subcutaneous fat, kg	**0**.**272**	<**0**.**001**	0.117	0.083	**0**.**156**	**0**.**015**
Marbling fleck area, %	**0**.**187**	**0**.**008**	−0.020	0.775	0.022	0.746
Number of marbling flecks	**0**.**191**	**0**.**006**	0.053	0.449	−0.031	0.645

Normalized protein values of SCF showed low but significant correlations with body weight and fat deposition-related traits (Table 1). Furthermore, we found a significant correlation between the RBP4 levels in plasma and those measured in SCF, with r = 0.35 (P < 0.001) at slaughter.

### Weak relationships between RBP4 protein abundance and concentrations of hormones and fatty acids in plasma

Insulin, glucagon, and leptin levels were measured in the plasma of F_2_-crossbred bulls at 8 months and at 18 months of age. The limited availability of samples resulted in sample sizes from 93 to 243 for correlation analyses. These numbers are given in respective figures, tables or in the text. Correlation coefficients between plasma insulin, glucagon, and leptin, concentrations at both ages and carcass traits are listed in Supplemental Table 3. Similar relationships as those recorded for RBP4 were observed between insulin and carcass fat traits. This corresponded to the weak correlation between RBP4 and insulin levels at 8 months of age (r = 0.20, n = 93, P = 0.049). However, there were no correlations observed between RBP4 protein amounts and other hormone levels at either age. There was a significant correlation between leptin level at 8 months and at 18 months of age (r = 0.50; P < 0.001; n = 232). Furthermore, insulin and glucagon levels at 8 months of age were correlated with fasting glucose levels at 8 months of age (r = 0.26, n = 101, P = 0.008 and r = −0.20, n = 228, P = 0.003, respectively).

A weak correlation was also observed between plasma RBP4 at slaughter and the respective total plasma lipid concentration (r = 0.13, n = 216, P = 0.05), but no correlations with the percentages of individual fatty acids exist (data not shown).

### Differences in plasma hormone concentrations and fatty acid composition in bulls with high or low carcass fat deposition

The hormone levels determined at 8 months of age and at slaughter at 18 months of age are presented for the two groups of bulls in Table 2. Similar levels (P > 0.05) of leptin were recorded for both groups of bulls at both ages, for glucagon at 8 months of age and for insulin at 18 months of age. The concentrations of glucagon at 18 months of age and for insulin at 8 months of age were increased in the HCF bulls (P = 0.013 and 0.019, respectively).

**Table 2:** Hormone concentrations in the plasma of crossbred bulls with high (HCF) or low carcass fat (LCF) at 8 months of age and at slaughter with 18 months of age.

Item	Age, months	HCF			LCF			P - value
		n	LSmean	SE	n	LSmean	SE	
Leptin, ng/mL	8	20	3.78^a^	0.43	18	3.77^a^	0.45	0.983
	18	20	9.65^b^	1.44	17	7.82^b^	1.54	0.391
Glucagon, pg/mL	8	20	66.52^a^	3.53	18	62.06	3.72	0.309
	18	17	177.57^b^	18.76	17	106.93	19.26	0.013
Insulin, µU/mL	8	20	13.68^a^	1.15	17	9.52^a^	1.25	0.019
	18	13	25.50^b^	3.15	16	20.02^b^	3.15	0.226

^a,b^ indicates significant differences between ages within a group (P < 0.05).

The plasma lipid content (Table 3) was not different between the groups (P = 0.96), but the fatty acid profiles indicated some remarkable differences (Table 3). Plasma of the LCF group contained a higher proportion (P = 0.029) of saturated fatty acids (SFA) and a lower proportion (P = 0.026) of polyunsaturated fatty acids (PUFAs), primarily based on lower values for linoleic acid (C18:2*n*-6). In the case of SFA, the C12:0, C14:0 and C15:0 proportions in the plasma of the HCF group were significantly lower compared with the LCF group. The main fatty acid in bull plasma was C18:2*n*-6 up to a proportion of 46% of the total fatty acids. The sum of monounsaturated fatty acids (MUFA) was not different between both groups, but *trans* vaccenic acid (C18:1*trans*-11) and C15:1*cis*-10 levels were significantly higher in the HCF bulls.

**Table 3:** Fatty acid profile in plasma of crossbred bulls with high (HCF, n = 20) or low carcass fat (LCF, n = 18) at slaughter with 18 months of age.

Fatty acid, %	HCF		LCF		P - value
	LSmean	SE	LSmean	SE	
C12:0	0.06	0.01	0.13	0.02	**0**.**005**
C14:0	0.74	0.06	1.15	0.12	**0**.**002**
C15:0	0.55	0.03	0.82	0.05	<**0**.**001**
C16:0	10.95	0.25	11.37	0.34	0.311
C17:0	0.92	0.03	1.06	0.05	**0**.**010**
C18:0	14.73	0.19	14.89	0.22	0.574
C20:0	0.84	0.17	1.16	0.14	0.170
C22:0	1.08	0.07	1.19	0.06	0.229
*Sum SFA*	32.95	0.88	35.99	0.76	**0**.**015**
C15:1*cis*-10	0.24	0.02	0.34	0.04	**0**.**023**
C16:1*cis*-9	2.40	0.09	2.68	0.11	0.056
C17:1*cis*-9	0.25	0.02	0.36	0.05	**0**.**033**
C18:1*trans*-11	0.58	0.03	0.71	0.06	**0**.**028**
C18:1*cis*-9	6.96	0.42	7.77	0.58	0.261
C18:1*cis*-11	0.85	0.05	0.88	0.04	0.702
C20:1*cis*-11	0.05	0.01	0.06	0.01	0.720
*Sum MUFA*	11.78	0.50	13.31	0.69	0.077
C18:2*n*-6	45.70	1.06	41.35	1.50	**0**.**021**
C18:3*n*-3	2.74	0.14	2.67	0.15	0.753
C20:5*n*-3	0.22	0.07	0.19	0.02	0.700
C20:4*n*-6	3.09	0.19	3.15	0.20	0.844
C22:4*n*-6	0.63	0.05	0.66	0.06	0.691
C22:5*n*-3	0.48	0.04	0.54	0.05	0.311
C22:6*n*-3	0.13	0.03	0.12	0.02	0.958
*Sum PUFA*	55.19	1.15	50.68	1.35	**0**.**015**
*Sum n-3 PUFA*	3.57	0.16	3.53	0.19	0.887
*Sum n-6 PUFA*	51.63	1.10	47.15	1.42	**0**.**016**
Fat content, %	0.34	0.02	0.35	0.02	0.630

Sum SFA: 10:0 + 11:0 + 12:0 + 13:0 + 14:0 + 15:0 + 16:0 + 17:0 + 18:0 + 20:0 + 21:0 + 22:0 + 23:0 + 24:0,

Sum MUFA: 14:1*cis*-9, 15:1*cis*-10, 16:1*cis*-9, 17:1*cis*-10, 18:1*trans*-11, 18:1*cis*-9, 18:1*cis*-11, 22:1*cis*-13, 24:1*cis*-15.

Sum PUFA: 18:2*tr*-9, *tr*-12 + 18:2*n-*6 + 18:3*n-*3 + 18:4*n*-3 + 20:3*n*-6 + 20:4*n-*6 + 20:5*n-*3 + 22:1 + 22:4*n-*6 + 22:5*n-*3 + 22:6*n*-3 + c9, tr11CLA + 18:3*n*-6 + 20:2*n*-6 + 20:3*n*-3 + 22:2*n*-6.

Sum *n*-3 PUFA: 20:3*n*-3 + 22:6*n*-3 + 22:5*n*-3 + 20:5*n*-3 + 18:4*n*-3 + 18:3*n*-3,

Sum *n*-6 PUFA: 22:2*n*-6 + 20:2*n*-6 + 18:3*n*-6 + 22:4*n*-6 + 20:3*n*-6 + 18:2*n*-6 + 20:4*n*-6.

### Similar glucose tolerance in bulls with high or low carcass fat deposition

**Figure 6:**
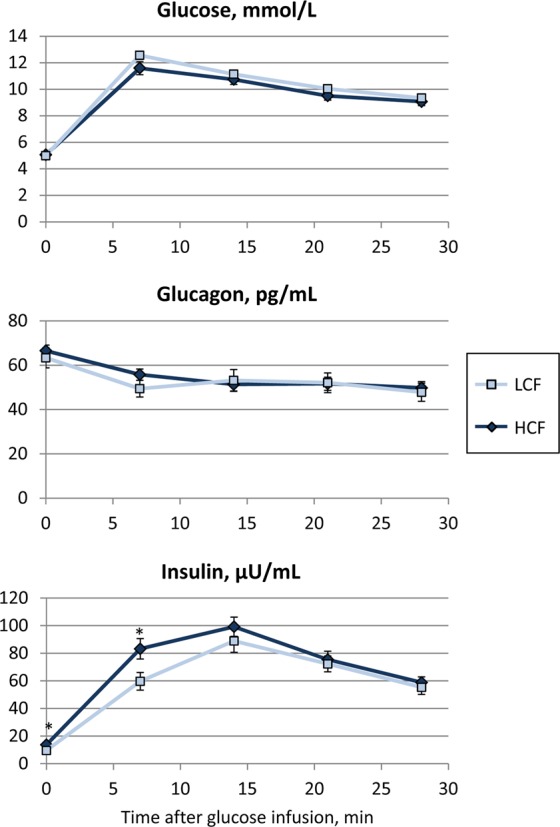
Plasma glucose, glucagon, and insulin concentrations during the glucose tolerance test in high carcass fat (HCF, n = 20) and low carcass fat (LCF, n = 18) bulls at 8 months of age. * indicate significant differences between groups with P < 0.05.

Bulls were subjected to a glucose tolerance test at an age of 8 months. The course of the plasma glucose and glucagon concentrations during this test was similar between both groups of bulls (Fig. 6). There was no difference between groups in basal plasma glucose concentrations after 12 h of fasting (P = 0.71). The glucose concentration increased (P < 0.001) within 7 min of glucose infusion and decreased thereafter (P < 0.001). Plasma glucagon concentration decreased within 7 min after infusion and remained almost constant (P > 0.2) thereafter in both groups. Insulin increased faster in the HCF bulls and reached significantly higher values (P = 0.017) after 7 min of glucose infusion (Fig. 6). However, the peak values and the following values were not different between the groups (P > 0.1).

## Discussion

Vitamin A (retinol and its metabolic products) is of crucial importance for many physiological processes, such as vision, growth, development, reproduction, and lipid and glucose metabolism, mostly by activation of the transcription factors (reviewed by Saeed *et al*.^24^). Studies in cattle described an increased intramuscular fat deposition as a result of decreased vitamin A supply^17,20^. Consequently, the abundance and availability of RBP4, as the sole transporter of vitamin A in the organism, may indirectly affect the abovementioned processes. The results in humans and rodent models established links between RBP4 expression and obesity, diabetes and other metabolic diseases. However, there are many controversial discussions regarding the mechanism of action and the origin of RBP4 in different species (reviewed by Kotnik *et al*.^6^). In contrast, data on RBP4 in cattle are rare. We demonstrated here that *RBP4* is expressed at the mRNA and protein level in different adipose depots and the livers of adult cattle. *RBP4* mRNA is stably expressed in SCF, OF, PF, and IF, in concordance with Locher *et al*.^25^, and in the liver and skeletal muscle tissue. Western blot data revealed a similarly robust protein expression in all tissues except in skeletal muscle. This is in accordance with data in humans and mice indicating liver cells and adipocytes as major sources of RBP4 expression^26,27^. Thompson *et al*.^8^ showed that the liver is the exclusive source of circulating RBP4 in mice, and adipocyte expression of RBP4 seemed not to result in secretion, suggesting an auto- or paracrine function for adipocyte-derived RBP4. This in turn contradicts the hypothesis that adipose depots, especially visceral fat, are major contributors to circulating RBP4 with increasing obesity^27^. We measured significantly higher plasma RBP4 levels in cattle with high compared to low body fat deposition at an age of 8 months as well as 18 months. Since there were no different protein levels of RBP4 in the livers of HCF and LCF bulls at an age of 18 months, we speculate that subcutaneous and intestinal fat could be major contributors to increased plasma RBP4 in HCF bulls. This assumption is supported by the positive correlation between RBP4 levels in plasma and in subcutaneous fat in our study. Chen *et al*.^28^ identified RBP4 as secreted from rat adipocytes in a proteome study, further supporting adipocytes as a possible source of circulating RBP4. However, a significant positive correlation between liver weight and plasma RBP4 (r = 0.25, P < 0.001) does not preclude that the larger amounts of liver tissue in HCF bulls may have contributed to the increase in plasma RBP4.

We observed significant correlations between the levels of plasma RBP4 at an age of 8 months and a number of fatness traits derived at slaughter in the total population. These relationships disappeared completely when considering RBP4 at an age of 18 months, thus indicating a time-dependent involvement of circulating RBP4 in fat deposition. Interestingly, the time window for the intensive formation of intramuscular adipocytes is supposed to end at the age of 8 months in cattle^29^ and may explain the positive relationship of plasma RBP4 with the marbling traits at this age. Higher levels of circulating RBP4 at 8 months than at 18 months of age suggest involvement in adipose tissue development instead only being a result of increased adipose tissue mass.

Our results of immunohistochemistry suggest the adipocyte as an important cell type for RBP4 protein storage in cattle, as was shown in humans and mice earlier^5,8^. However, further cell types may contribute to RBP4 expression in adipose tissues, as indicated by immuno-staining of the cells located between adipocytes. In contrast, muscle fibres were generally negative for RBP4. Exclusively few cells in the connective tissue between muscle fibres revealed a RBP4-positive signal. This may explain the discrepancy between the expression of *RBP4* mRNA in bovine skeletal muscle with highly sensitive RT-qPCR and the failure to detect the protein by less sensitive western blots in the same tissue.

Liver tissue is considered the main storage for RBP4 and even as an exclusive source of circulating RBP4 in rodents^3,8,26^. Consistent with these findings, we noticed high protein abundance in the liver tissue of cattle. Surprisingly, immunohistochemistry revealed strong RBP4 signals in cells, which were mainly identified as stellate cells because of their typical auto-fluorescence, but not in hepatocytes. Macrophages have been shown to be a cell type with a significant expression of RBP4 *in vitro*^11^. The counterstaining of bovine liver tissue with the macrophage marker CD163 showed a few RBP4-positive but mainly negative macrophages, demonstrating that the latter cells do not contribute much to the RBP4 abundance of bovine liver. Collectively, our data demonstrate a high expression of RBP4 protein in bovine liver and different adipose tissue depots as described in humans and rodents^5,26^, but different localization of RBP4 in the liver and no difference in RBP4 liver levels between fat and lean cattle. Instead, adipose depots seem to play a larger role in the varying amounts of circulating RBP4 observed in both groups of cattle. This in turn supports the consideration of RBP4 as an adipokine^5^.

Transcriptome studies in bovine muscle with varying intramuscular fat content and a proteome study of bovine subcutaneous fat, before and after 3 months of fattening, have identified *RBP4* as differentially expressed and led to the conclusion that *RBP4* is involved in lipid metabolism and fat deposition in cattle^15,16,30^. This is consistent with findings in humans, where increased levels of circulating RBP4 were correlated with fat deposition, and elevated protein amounts were found in the adipose tissue of obese subjects^27,31–33^. A recent study by Perduca *et al*.^9^ demonstrated the ability of RBP4 to bind fatty acids. It was suggested that this function is restricted to the retinol-unbound form of RBP4 (apo). The authors concluded that RBP4 should be considered a lipid-binding protein rather than a specific retinol transporter. If RBP4 binds and transports mainly SFAs^9^, this could contribute to the observed significant differences in plasma fatty acid profiles of bulls with high and low fat deposition in our study. However, the proportions of saturated fatty acids were decreased in the plasma of HCF bulls (less myristic and pentadecanoic acid), whereas C18:2*n*-6 was significantly increased, leading to a significantly higher proportion of PUFAs. There was no difference in the total lipid content of the plasma of both groups; thus, a clear relationship between different concentrations in circulating RBP4 and the fatty acids in plasma seems to be unlikely. Nevertheless, RBP4 should be considered in its new function^9^ in further studies.

In humans, the increased serum RBP4 levels were also associated with obesity and insulin resistance^5^. The study of Graham *et al*.^34^ showed that the elevation of serum RBP4 occurred before diabetes development and that it was correlated with insulin resistance and cardiovascular risk factors in subjects with obesity, type 2 diabetes, impaired glucose tolerance and in non-obese, non-diabetic subjects with a strong family history of type 2 diabetes. They found that the level of circulating RBP4 is linked more specifically with insulin resistance than that of other adipokines such as leptin and adiponectin. Since adipose tissue is considered a contributor to elevated serum RBP4 levels in the state of insulin resistance, we tested the glucose tolerance of the bulls. We found no differences in glucose clearance or glucagon levels between HCF and LCF bulls and only slightly increased insulin levels in HCF bulls during the first minutes of the test, indicating that even the bulls with higher carcass fat were in a metabolic healthy state at an age of 8 months. Increased glucagon and similar insulin levels in HCF compared to LCF bulls at slaughter are signs of an increased gluconeogenesis but still do not indicate a diabetic state in these bulls. Leptin was not different at 8 months of age; it increased during growth but did not result in significant differences at slaughter between HCF and LCF bulls. This underlines that the intensive fattening system, which is widely used in beef production, does not evoke metabolic diseases. Even bulls with the highest carcass fat deposition from our population were not obese in terms of a pathological condition.

The results of our study suggest that the protein expression of *RBP4* in adipose tissues contributes to circulating RBP4 in cattle. We found significant relationships between body composition and RBP4 levels in cattle as described for other species. Further studies are required to elucidate how RBP4 is involved in bovine adipose tissue development. Blocking the insulin stimulated phosphorylation of the insulin receptor, as shown in primary human adipocytes^35^, could be a possible mechanism.

In summary, we provided data of *RBP4* gene expression on mRNA and protein level as well as cellular localization of RBP4 in cattle. Our results indicate differences in localization (and possibly secretion) of RBP4 in cattle compared to those reported for rodents. Circulating RBP4 is increased already at juvenile age in bulls, with elevated fat deposition at a later age, even without any sign of metabolic disturbances. Consequently, increased circulating RBP4 may be an indicator for higher fat accumulation in cattle.

## Materials and Methods

### Animals and sampling

This study involved 246 F_2_-generation bulls of a Charolais × Holstein cross, raised under standardized feeding and housing conditions and slaughtered at 18 months of age in the abattoir of the Leibniz Institute for Farm Animal Biology^21,36^. Animal care and experimental procedures followed the guidelines of the German Law of Animal Protection. The protocols were approved by the Animal Protection Board of the Leibniz Institute for Farm Animal Biology as well as by the Animal Care Committee of the State Mecklenburg-Western Pomerania, Germany (State Office for Agriculture, Food Safety and Fishery; LALLF M-V/TSD/7221.3-2.1-010/03).

The bulls were extensively phenotyped after slaughter, including carcass composition, meat quality, chemical analyses, muscle structure and marbling, as described previously by Liu *et al*.^23^. Blood samples were taken as described by Weikard *et al*.^37^ at an average age of 8 months and at slaughter. Briefly, blood was collected from the left jugular vein into EDTA tubes (Sarstedt AG & Co, Nümbrecht, Germany) by a standardized procedure at 7:30 AM after a fasting period of 12 h. Plasma was obtained from blood samples by centrifugation within 30 min after sampling. Plasma samples were stored at −80 °C until further use. Tissue samples from subcutaneous fat (SCF), perirenal fat (PF), omental fat (OF), intestinal fat (IF), the liver, and M. longissimus dorsi (MLD) were taken immediately after slaughter, frozen in liquid nitrogen and stored at −80 °C.

Two groups of bulls with either high or low carcass fat were retrospectively assigned to enable comparisons between animals with extreme differences in body composition. Bulls were sorted according to their carcass fat mass, and each of the 20 animals with the highest and lowest mass were assigned to the high carcass fat group (HCF; 94.2 ± 6.4 kg) and low carcass fat group (LCF; 36.0 ± 6.4 kg), respectively. Two bulls from the LCF group were excluded from further analyses due to health problems. Thus, the LCF group was comprised of 18 bulls. Higher (P < 0.01) concentrate consumption (HCF: 7.5 ± 0.6 kg/d vs. LCF: 5.9 ± 0.7 kg/d), residual feed intake (HCF: 7.0 ± 4.8 vs. LCF: −3.5 ± 6.1), and feed conversion ratio (HCF: 70.2 ± 3.6 MJ ME/kg vs. LCF: 65.4 ± 6.2 MJ ME/kg) were recorded for bulls with higher carcass fat deposition, using calculations described by Widmann *et al*.^36^.

### Glucose tolerance test and plasma hormone levels

Bulls were subjected to an intravenous glucose tolerance test (GTT) at the age of 8 months as described by Lahann *et al*.^38^. Briefly, glucose (1 g/kg of BW^0.75^) was infused into the jugular vein via a jugular cannula after a period of 12 h fasting. Blood samples were taken before (0) and 7, 14, 21, and 28 min after glucose infusion. Blood samples for hormone (insulin, glucagon, leptin) measurements were collected in tubes with potassium-EDTA (1.6 mg/mL of blood, Monovette, Sarstedt). Samples were centrifuged at 1,500 × g for 20 min at 4 °C and supernatants were aliquoted and stored at −80 °C until analysed.

Plasma glucose was analysed spectrophotometrically (HORIBA ABX SAS, Montpellier, France) by a kit (#A11A01667; Axon Lab AG, Baden, Switzerland) using the hexokinase method. Insulin was measured in the plasma by RIA using a porcine kit (PI-12K, Linco Research, St. Charles, MO; as described by Bellmann *et al*.^39^). A standard curve was prepared at concentrations from 2 to 200 μU/mL. Cross-reactivity with bovine insulin was 90%. Previous findings have shown that this assay exhibited good linearity for concentrations in the range of 25 to 100 μU/mL in bovine plasma samples. The sensitivity of the insulin RIA (the lowest detectable level) was 2 µU/mL. Intra- and interassay coefficients of variation (CV) were 8.2 and 4.3%, respectively. Glucagon was measured by RIA using a kit from Linco (GL-32K, Linco Research) as described by Hammon *et al*.^40^. Intra- and interassay CV were 9.6 and 5.8%, respectively. The assay is specific for the determination of pancreatic glucagon in serum or plasma in most mammals. Cross-reactivity to oxyntomodulin, which is the primary gut glucagon, is less than 0.1%.

The concentrations of leptin were determined by an in-house developed, competitive ELISA with biotinylated ovine leptin as tracer as described by Sauerwein *et al*.^41^. The rabbit antiserum was generated using a combination of recombinant ovine leptin and peptides out of the bovine leptin sequence. Accuracy was confirmed by demonstrating parallelism between standard curves and serial dilutions of sera from various species, including cattle. The limit of detection was 0.3 ng/mL; the intra- and interassay coefficients of variation were 6.3 and 13.9%, respectively.

### Plasma lipid extraction and fatty acid analysis

Plasma samples, 1500 µL, were added dropwise to 8 mL chloroform/methanol (2:1, v/v) at room temperature. The solution contained C19:0 as an internal standard. The detailed sample preparation procedure has recently been described by Dannenberger *et al*.^42^. Briefly, all of the solvents contained 0.005% (w/v) of t-butylhydroxytoluene (BHT) to prevent the oxidation of PUFAs. The extraction mixtures were stirred two times for 15 min and stored at 5 °C for 18 h in the dark and subsequently washed with 0.02% CaCl_2_ solution. After centrifugation (2500 rpm, 5 min), the organic phase was dried with Na_2_SO_4_ and K_2_CO_3_ (10:1, wt/wt), and the solvent was subsequently removed under a gentle nitrogen stream at room temperature. The total lipid contents were stored at −18 °C until transmethylation of fatty acids. The lipid extracts were redissolved in 150 µL of toluene for methyl ester preparation. Next, 1 ml of 0.5 M sodium methoxide in methanol was added to the samples, which were shaken in a 60 °C water bath for 10 min. Subsequently, 0.5 mL of 14% boron trifluoride (BF_3_) in methanol was added to the mixture, which was then shaken for an additional 10 min at 60 °C. Saturated NaHCO_3_ solution (2 mL) was added, and the fatty acid methyl esters (FAMEs) were extracted three times in 2 mL of *n*-hexane. The combined *n*-hexane extract was dried with Na_2_SO_4_ and K_2_CO_3_ (10:1, wt/wt) and, after filtration, reduced to dryness using a vacuum centrifuge (2000 rpm, 30 °C, 30 min). The FAMEs were resuspended in 100 µL of *n*-hexane and stored at −18 °C until they were used for gas chromatography (GC) analysis.

The fatty acid analysis of the bull plasma lipids was performed using capillary GC with a CP-Sil 88 CB column (100 m × 0.25 mm, Agilent, Santa Clara, CA, United States) that was installed in a PerkinElmer gas chromatograph CLARUS 680 with a flame ionisation detector and split injection (PerkinElmer Instruments, Shelton, United States). The detailed GC conditions were recently described by Dannenberger *et al*.^43^. Briefly, the initial oven temperature was 150 °C, which was held for 5 min; subsequently, the temperature was increased to 175 °C and then to 200 °C at a rate of 2 °C/min and held for 10 min. Finally, the temperature was increased to 225 °C at a rate of 1.5 °C/min and held for 25 min. Hydrogen was used as the carrier gas at a flow rate of 1 mL/min. The split ratio was 1:20, and the injector and detector were set at 260 °C and 280 °C, respectively. The quantification of fatty acids was done by the use of C19:0 as the internal standard. For the calibration procedure the reference standard mixture ‘Sigma FAME’ (Sigma-Aldrich, Deisenhofen, Germany), the methyl ester of C18:1*cis*-11, C22:5*n*-3 and C18:2*cis*-9, *trans*-11 (Matreya, PA, USA), C22:4*n*-6 (Sigma-Aldrich, Deisenhofen, Germany) and C18:4*n*-3 (Larodan, Limhamn, Sweden) were used. The five-point calibration of single fatty acids ranged between 16 and 415 mg/mL and was checked after the GC analysis of five samples.

### RNA isolation, cDNA synthesis, and qRT-PCR

The protocols for RNA isolation from adipose tissues, muscle and liver are described in detail by Schering *et al*.^44^. Total RNA was quantified by a NanoDrop ND-1000 spectrophotometer (Peqlab, Erlangen, Germany). The integrity of the RNA was determined with an Experion Automated Electrophoresis System using the RNA StdSens analysis chip (Bio-Rad, Munich, Germany). The samples of RNA with an RQI (RNA quality indicator) value above 7 were considered as qualified for further use. First strand cDNA was synthesized from 100 ng total RNA of each sample in 20 µL reaction volume according to manufacturer’s instructions (iScript cDNA Synthesis Kit, Bio-Rad).

Quantitative reverse transcription PCR with iQ detection system (Bio-Rad) was performed in duplicate as previously described^44^ to analyse expression levels of *RBP4*. Briefly, primers (*RBP4* NM_001040475.2, for: TGAGCAGCTTCCGAGTCAAG, rev: GCGACGATGTTGTCTTGCAG) were designed with Primer 3web (Version 4.0.0, http://primer3.ut.ee/) and synthesized by Sigma-Aldrich. Each reaction contained 10 ng cDNA template, 0.2 µM of the respective forward and reverse primers, and 5 µL SYBR Green Supermix (Bio-Rad) in 10 µL reaction volumes. All reactions were carried out with following conditions: initial denaturation of 95 °C for 3 min and 45 cycles of 95 °C for 10 s, 60 °C for 30 s, 70 °C for 45 s. The amplicon specificity was confirmed by melting curve analysis and sequencing. The expression values for *RBP4* were normalized as described by Liu *et al*.^23^ to two reference genes in each tissue: beta-2-microglobulin (*B2M*) and ubiquitously expressed transcript (*UXT*) for adipose tissues; *UXT* and ribosomal protein S9 (*RPS9*) for liver tissue; topoisomerase II beta (*TOP2B*) and *B2M* for muscle tissue. Crossing point (C_P_) values were adjusted manually in the iQ5 software (Version 2.1.97.1001, Bio-Rad) to obtain comparable values for the standard curves on each plate. The efficiency of amplifications was calculated from the standard curves with serial dilutions (1:1, 1:10, 1:50, 1:100, 1:500). The efficiency-corrected ΔΔC_p_ method^45^ was applied for normalizations of C_P_ values. The fold change between the two groups was determined with the REST algorithm (Version 2.0.13, QIAGEN, Hilden, Germany). Normalized relative quantities of mRNA (NRQ^46^) were used for correlation analyses.

### Western blotting

Bovine plasma RBP4 was quantified by western blot analysis after albumin removal with Aurum Affi-Gel Blue (BioRad, Munich, Germany) in 20 µg protein per lane. Additionally, the original plasma samples were analysed (0.5 µl per lane) together with a dilution series of recombinant bovine RBP4 (Cloud-Clone, USC-RPA929BO01, purchased from Biozol, Eching, Germany) using 40, 20, 10, 5, and 2.5 ng per lane to determine the absolute plasma concentration (Supplemental Fig. 5). Total protein of adipose tissues, liver, and muscle tissue was isolated with CelLytic MT lyses reagent (Sigma-Aldrich, Munich, Germany) and protease inhibitor according to manufacturer’s instructions. Protein mixed with the loading buffer was denatured at 95 °C for 5 min and then loaded on Criterion TGX 12% gels (Bio-Rad). The molecular weight of the protein bands was determined by two molecular weight markers (PageRuler, Thermo Scientific; Triple Color Protein Standard III, SERVA). After electrophoresis, the proteins were transferred to a polyvinylidene difluoride (PVDF) membrane (Tans-Blot Turbo transfer pack, Bio-Rad) with a semi dry blotter (Trans-Blot, Bio-Rad). Proper transfer of the protein to membranes was verified by Coomassie staining (Brilliant Blue R-250, Carl Roth) or analysing the 647 nm image when using the Smart Protein Layers (SPL) Western Kit Red (PR913-R, NH Dyagnostics GmbH, Halle, Germany) for RBP4 quantification in plasma samples. Membranes were blocked for 1 h with 10% Roti-Block (Carl Roth) in Tris-buffered saline (TBS). Then, the membranes were incubated with a primary antibody against RBP4 overnight at 4 °C. Two different polyclonal antibodies against RBP4 were used, one was produced by Pierce (Thermo Fisher Scientific, Huntsville, USA, custom made, bovine specific) and the other was from Biorbyt (orb136234, purchased from Biozol, Eching, Germany). Recombinant bovine RBP4 (Cloud-Clone) was produced in *E. coli* with N-terminal his-tag and was used as a positive control and for the blocking of specific binding of the RBP4 antibodies. For specificity tests, two parallel blots were incubated with the antibody or with the antibody blocked with the recombinant protein before incubation. All membranes were incubated with HRP conjugated Trueblot rabbit IgG (1:25,000; eBioscience, Frankfurt, Germany) secondary antibody. The antibody label was detected with highly sensitive chemiluminescence substrate (SuperSignal West Femto Substrat, Thermo Scientific). Chemiluminescence was recorded with a Chemocam HR-16 imager (INTAS, Göttingen, Germany) and quantified with Labimage 1D software (Kapelan Bio-Imaging, Leipzig, Germany). All samples were measured at least in duplicates on separate blots.

### Immunohistochemical analysis

Frozen bovine tissues were cryo-sectioned (thickness liver: 10 µm; MLD: 12 µm) using a Leica CM3050 S (Leica, Bensheim, Germany) cryostat microtome. The air dried sections were fixed with 4% paraformaldehyde for 10 min, 2 × 5 min washed with PBS and permeabilized for 10 min with PBS including 0.1% of TritonX100 (PBST). Liver samples were treated with filtrated Sudan Black (0.1% in 70% ethanol, Waldeck GmbH & Co. KG) for 30 min and washed twice with PBS to reduce auto-fluorescence. After blocking with 10% goat serum in PBST for 15 min, sections were incubated with the Biorbyt antibody against RBP4 (1:100 in PBST) for 1 h at room temperature in a humidity chamber. For double labelling experiments, a monoclonal mouse CD163 antibody was added (1:100, AbD Serotec, Puchheim, Germany) to the incubation solution. Goat anti-rabbit or mouse IgG secondary antibodies labelled with Alexa Fluor 488 (Molecular Probes, Eugene, USA) or with MFP590 (MoBiTec) were used for the detection of the specific binding of the primary antibodies. Nuclei were counterstained with 1 µg/mL Hoechst 33258 (Sigma-Aldrich, Munich, Germany). Slides were covered with the appropriate cover-slips using ProLong Diamond Antifade Mountant (Thermo Scientific). Slides used as negative controls were either incubated without a primary antibody or with the primary antibody after blocking with recombinant RBP4. No unspecific binding of the secondary and the primary antibody was detected. Immunofluorescence was visualized on a Nikon Microphot SA fluorescence microscope (Nikon, Duesseldorf, Germany) equipped with a CC-12 high-resolution colour camera (OSIS, Muenster, Germany) and CELL^F imaging software (OSIS).

### Statistical analyses

Data were analysed with SAS statistical software (Version 9.3, SAS Inst., Cary, USA). Relationships were calculated by Pearson correlation coefficients using the CORR procedure in SAS. The normalized protein values of RBP4 in tissues were analysed with ANOVA using the MIXED procedure with fixed factor group and repeated factor technical replicate (with unstructured covariance structure). The plasma data was analysed with the MIXED procedure with fixed factors group, repeated factor age (with autoregressive covariance structure), and group × age interaction. Accordingly, the plasma hormone and glucose data of the glucose tolerance test was analysed with the MIXED procedure with fixed factors group, repeated factor time (with autoregressive covariance structure), and group × time interaction. The Tukey-Kramer-correction was used to control the Family-wise Error Rate (referred to as P_adj_). Differences were considered as significant if P_adj_ ≤ 0.05.

## Supplementary information

Supplementary Information

## Data Availability

Supplemental data are provided with this paper. Additional data and protocols are available on request via e-mail to the corresponding author.
