# Supplementary material for: Retinol binding protein 4 abundance in plasma and tissues is related to body fat deposition in cattle
Source: Sci Rep. 2019 May 30;9:8056. doi: 10.1038/s41598-019-44509-4 (PMC6542835; doi:10.1038/s41598-019-44509-4)
Supplement: Supplementary file 1 — Supplementary Information [file 41598_2019_44509_MOESM1_ESM.pdf]

Supplementary Information

Manuscript: Retinol binding protein 4 abundance in plasma and tissues is related to body fat deposition in cattle

By: Yinuo Liu, Elke Albrecht, Dirk Dannenberger, Harald M. Hammon, Christa Kühn, Helga Sauerwein, Runjun Yang, Zhihui Zhao, and Steffen Maak

Supplemental Table 1:

Selected phenotypic traits of two groups of F_2-generation bulls (Charolais × Holstein cross) slaughtered at 18 months of age (Liu et al.[22])

	Group				
	HCF		LCF		P -
Trait	LSmean	SE	LSmean	SE	value
Age, days	547	1	547	1	0.8886
Body weight, kg	712	11	626	16	<.0001
Average daily gain, kg	0.70	0.01	0.61	0.02	<.0001
Cold carcass weight, kg	402.8	6.7	358.5	10.1	0.0005
Liver, kg	7.87	0.19	6.30	0.24	<.0001
Meat, %	75.58	0.31	77.68	0.38	<.0001
Bones, %	13.74	0.13	16.09	0.40	<.0001
Tendons, %	2.43	0.05	2.72	0.07	0.0008
Carcass fat, kg	95.47	1.38	35.88	1.38	<.0001
Carcass fat, %	23.52	0.46	10.04	0.27	<.0001
Carcass protein, %	13.21	0.12	15.44	0.12	<.0001
Subcutaneous fat, %	8.25	0.28	3.44	0.14	<.0001
Intestinal fat, %	1.74	0.10	0.98	0.07	<.0001
Omental fat, %	2.85	0.13	1.73	0.10	<.0001
Perirenal fat, %	2.65	0.11	1.62	0.10	<.0001
M. longissimus weight, kg	7.85	0.19	7.80	0.28	0.3161
IMF[1] of M. longissimus, %	5.61	0.44	1.53	0.09	<.0001
Marbling fleck area percentage, %	8.01	0.59	2.31	0.24	<.0001
Number of marbling flecks	756	40	366	43	<.0001
Distance between marbling flecks, mm	1.71	0.03	2.23	0.17	0.0017

[1]IMF – intramuscular fat content

Supplemental Table 2: Raw C_p-values of *RBP4* mRNA in different bovine tissues

	LCF		HCF			Amplification
	LSMean	SE	LSMean	SE	P-value	efficiency
SCF	12.76^a	0.28	12.04^a	0.25	0.055	1.999
OF	16.27^b	0.27	15.32^b	0.25	0.010	2.042
PF	13.01^a	0.27	12.97^a	0.25	0.924	2.109
IF	17.81^c	0.27	17.00^c	0.25	0.029	1.938
Liver	10.72^d	0.27	10.31^d	0.25	0.270	1.998
MLD	19.37^e	0.27	18.82^e	0.25	0.136	1.934

$^{a\text{-}e}$ indicate significant differences between tissues within a group (P < 0.01).

Supplemental Table 3: Pearson correlation coefficients between traits of F_2-generation bulls at 18 months of age and the concentrations of insulin, glucagon, and leptin in plasma at 8 and 18 months of age. Bold numbers indicate significant correlations with P < 0.05.

Trait	Insulin		Glucagon		Leptin	
	8 months, n = 105	18 months, n = 203	8 months, n = 238	18 months, n = 217	8 months, n = 242	18 months, n = 234
Body weight, kg	0.016	0.114	-0.015	0.114	-0.091	-0.050
Liver, kg	0.048	**0.259**	-0.011	**0.189**	-0.116	0.003
Cold carcass weight, kg	-0.050	0.051	-0.011	0.060	-0.056	-0.032
Cold carcass fat, kg	**0.328**	**0.258**	**0.129**	**0.293**	0.027	**0.152**
Perirenal fat, kg	0.109	**0.317**	**0.223**	**0.427**	-0.082	0.055
Intestinal fat, kg	0.115	**0.259**	**0.189**	**0.339**	-0.052	0.042
Omental fat, kg	**0.214**	**0.334**	**0.141**	**0.407**	-0.044	0.111
Subcutaneous fat, kg	**0.334**	**0.258**	0.117	**0.308**	0.005	0.112
Marbling fleck area, %	0.146	**0.330**	0.100	**0.247**	-0.022	0.081
Number of marbling flecks	0.144	0.085	**0.242**	**0.203**	-0.043	-0.066

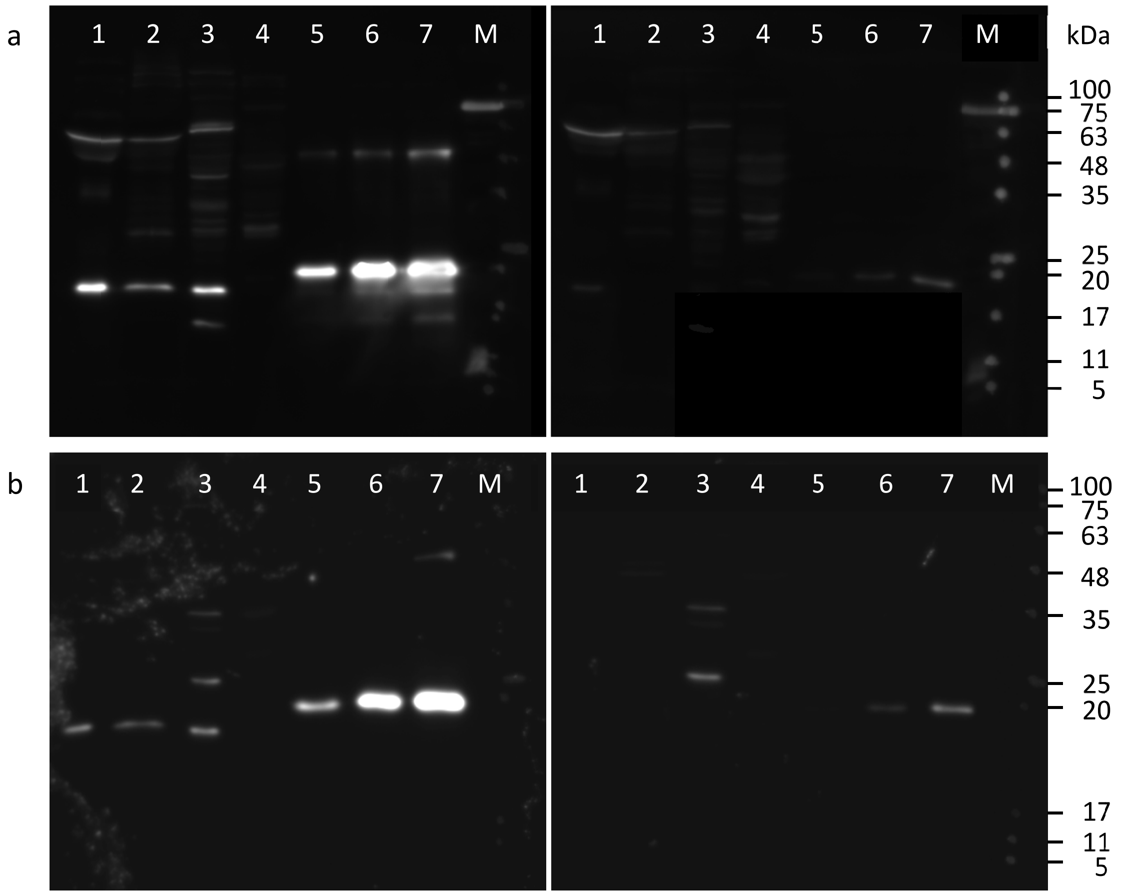

Supplemental Figure 1: Specificity test of the used RBP4 antibodies in western blot. Detection of RBP4 in bovine plasma and tissues, as well as bovine recombinant RBP4 using the antibody of Biorbyt (a) or Pierce (custom made, b) without (left) and with (right) blocking of specific binding by preincubation with recombinant RBP4. 1 – plasma; 2 –subcutaneous fat; 3 – liver; 4 – muscle; 5 – 12.5 ng recombinant RBP4; 6 – 25 ng recombinant RBP4; 7 – 50 ng recombinant RBP4; M – size marker.

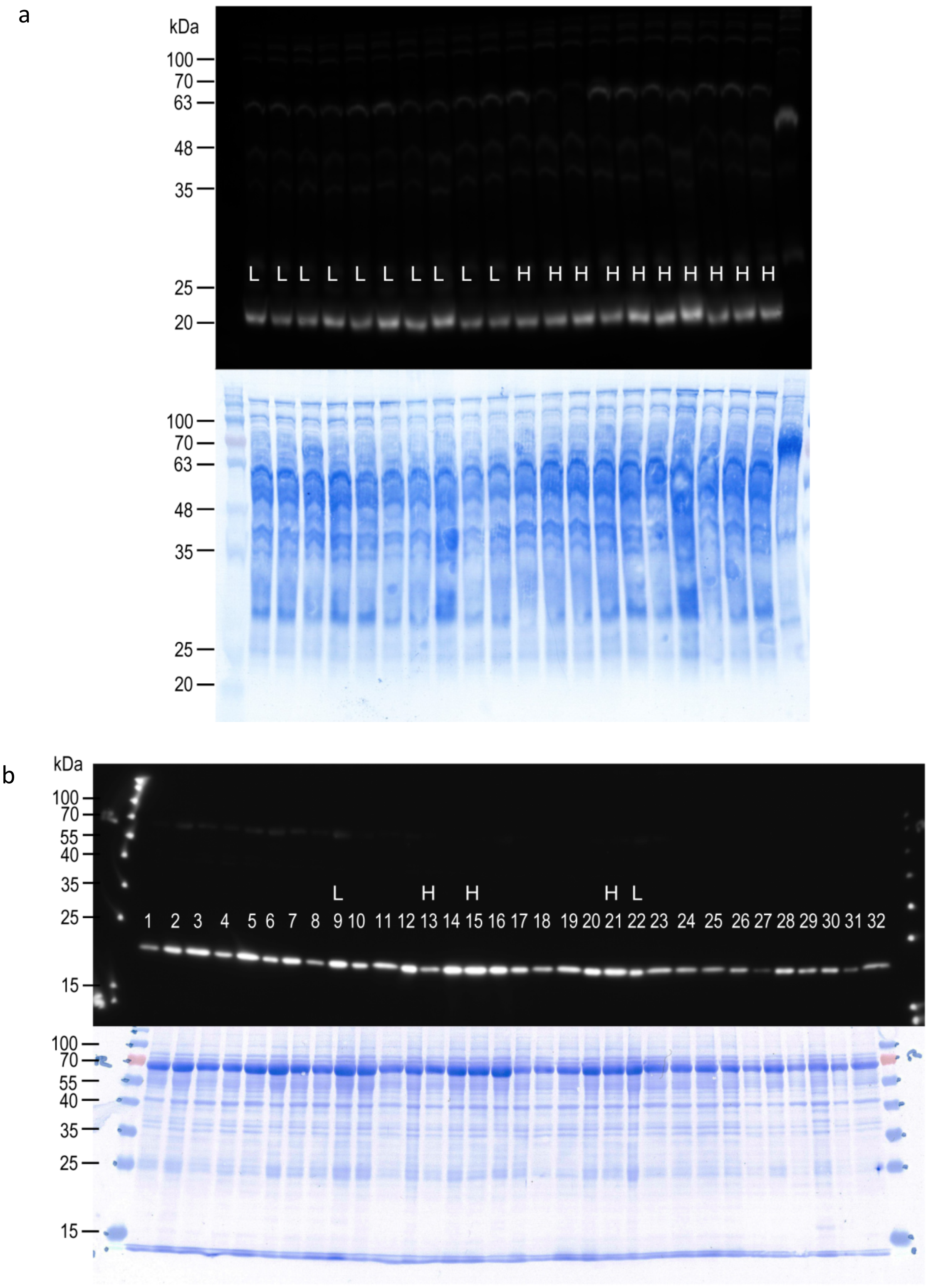

Supplemental Figure 2: Representative western blot image for RBP4 quantification in 20 liver samples (a) and 32 subcutaneous fat samples (b) of F_2-generation bulls (Charolais × Holstein cross) at 18 months of age. H and L indicate samples of bulls of the high and low carcass fat groups, respectively. The Coomassie stained blot image below was used to determine the amount of total protein in each lane for normalization.

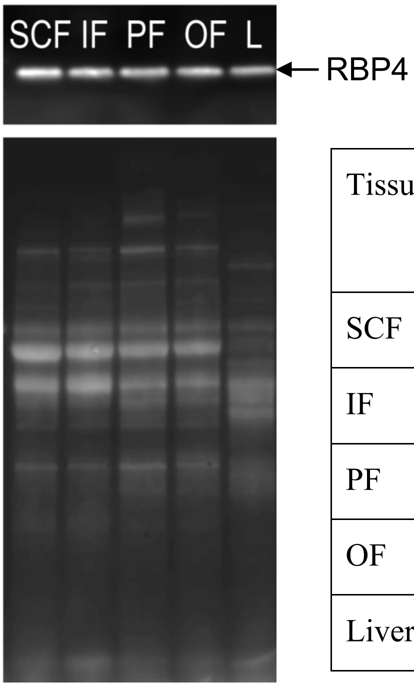

Tissue	Band volume of RBP4	Band volume of total protein	RBP4 / total Protein
SCF	13,353,760	58,155,272	0.230
IF	11,692,595	60,285,252	0.194
PF	10,749,767	52,957,840	0.203
OF	12,164,575	44,676,564	0.272
Liver	10,665,418	44,240,000	0.241

Supplemental Figure 3: Detection of RBP4 in five tissues of one bull with western blot. The upper image shows the chemiluminescence detection of RBP4. Total protein is shown below in the fluorescence image at 647 nm. It was stained using the SPL kit (NH DyeAgnostics GmbH, Halle, Germany). Protein of all tissues was equally extracted and treated. Ten µg of total protein was used in each lane. Respective band and lane volumes are presented in the table. SCF – subcutaneous fat; IF – intestinal fat; PF – perirenal fat; OF – omental fat; L – liver.

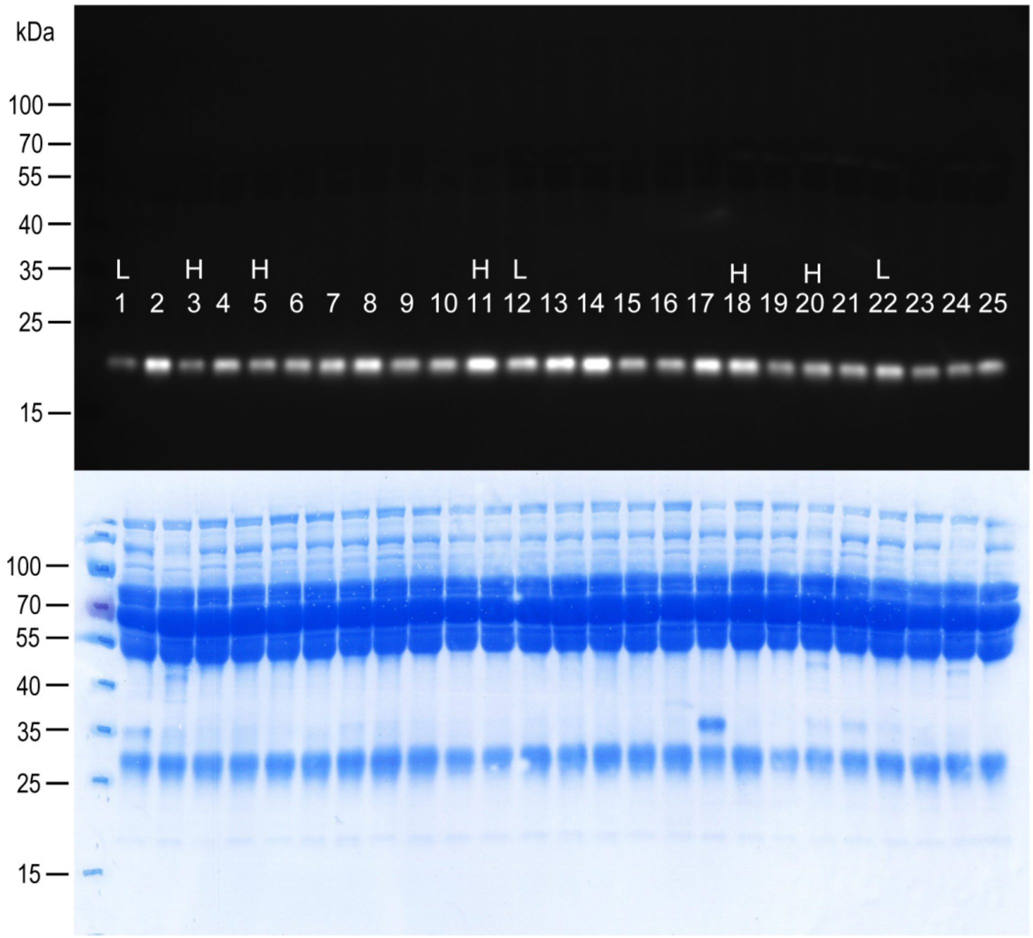

Supplemental Figure 4: Representative western blot image for RBP4 quantification in 25 plasma samples, after albumin depletion, of F_2-generation bulls (Charolais × Holstein cross) at 8 months of age. H and L indicate samples of bulls of the high and low carcass fat groups, respectively. The Coomassie stained blot image below was used to determine the amount of total protein in each lane for normalization.

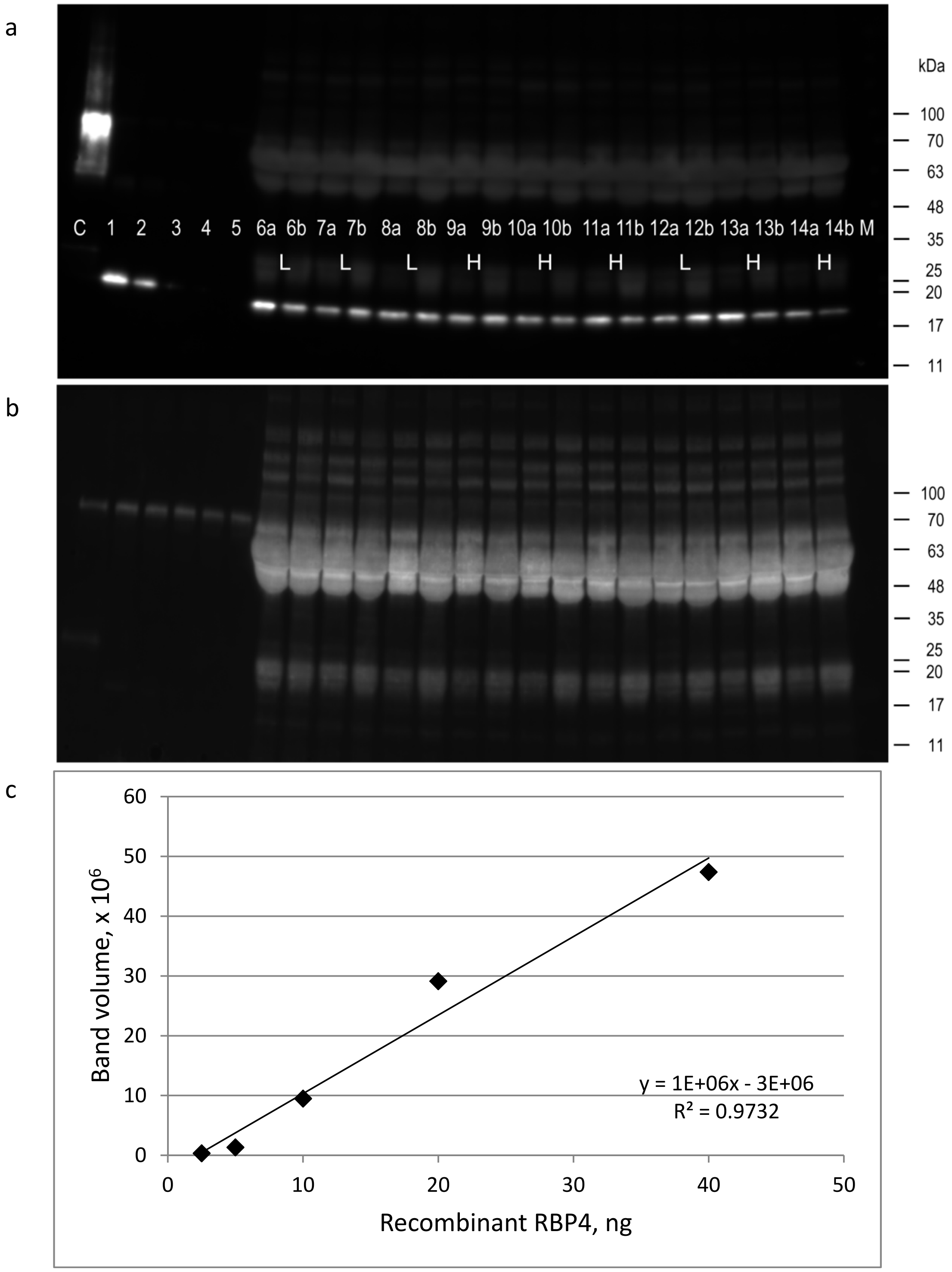

Supplemental Figure 5: Representative western blot image for RBP4 quantification in plasma samples of 9 F_2-generation bulls (Charolais × Holstein cross) at 8 months and 18 months of age. a: Chemiluminescence image of RBP4 detection. H and L indicate samples of bulls of the high and low carcass fat groups, respectively. C - calibrator, 1-5 recombinant RBP4 dilution series (40, 20, 10, 5, 2.5 ng), 6a-14b plasma samples taken at 8 (a) and 18 (b) months of age, respectively, M – marker. b: Total protein in each lane was determined in fluorescence image at 647 nm using the SPL kit (NH DyeAgnostics GmbH, Halle, Germany) for normalization. c: Standard curve used for calculation of protein amount in each lane.
